# Person-centered non-pharmacological interventions for agitation in dementia: a meta-analysis with subgroup analysis by behavioral subtypes

**DOI:** 10.3389/fneur.2026.1850987

**Published:** 2026-08-10

**Authors:** Ruilong Ye, Mengqing Lin, Lingjun Lei

**Affiliations:** 1 Department of Geriatrics (Respiratory and Critical Care Medicine), The Second People’s Hospital of Lishui, Lishui, China; 2 Department of Intensive Care Unit, The Second People’s Hospital of Lishui, Lishui, China

**Keywords:** agitation, behavioral subtypes, dementia, meta-analysis, non-pharmacological interventions, person-centered care

## Abstract

**Objective:**

This meta-analysis aimed to evaluate the overall efficacy of person-centered non-pharmacological interventions for agitation in dementia and to compare treatment effects across behavioral subtypes, specifically physical aggressive agitation, physical non-aggressive agitation, and verbal agitation.

**Methods:**

A systematic literature search was conducted in PubMed, Embase, Web of Science, and the Cochrane Central Register of Controlled Trials (CENTRAL) from database inception to March 2026. Randomized controlled trials (RCTs) evaluating person-centered non-pharmacological interventions for agitation in individuals with dementia were included. The primary outcome was agitation, with subgroup analyses performed for behavioral subtypes. Standardized mean differences (SMDs) with 95% confidence intervals (CIs) were calculated using random-effects models. Risk of bias was assessed using the Cochrane RoB 2.0 tool.

**Results:**

Six RCTs were included. Person-centered non-pharmacological interventions significantly reduced overall agitation compared with control conditions (SMD = −0.48, 95% CI: −0.84 to −0.13, *p* = 0.008; *I*^2^ = 81%). Subgroup analyses revealed a significant reduction in verbal agitation (SMD = −0.45, 95% CI: −0.73 to −0.17, *p* = 0.002; *I*^2^ = 0%), whereas reductions in physical non-aggressive agitation (SMD = −0.48, 95% CI: −1.06 to 0.11, *p* = 0.11; *I*^2^ = 84%) and physical aggressive agitation (SMD = −0.40, 95% CI: −1.63 to 0.83, *p* = 0.52; *I*^2^ = 86%) did not reach statistical significance. Substantial heterogeneity was observed for overall agitation and physical agitation subtypes, but not for verbal agitation.

**Conclusion:**

Person-centered non-pharmacological interventions significantly reduce overall agitation in individuals with dementia, with the most consistent effects observed for verbal agitation. The lack of significant effects on physical agitation subtypes, coupled with substantial heterogeneity, highlights the critical need for further research and intervention development specifically targeting these physical behaviors. These findings support the use of personalized interventions as a first-line strategy, particularly for verbal symptoms, while emphasizing the importance of further research to optimize interventions for physical agitation.

**Systematic review registration:**

https://www.crd.york.ac.uk/PROSPERO/view/CRD420261363075, identifier PROSPERO (CRD420261363075).

## Introduction

Dementia is a progressive neurodegenerative syndrome characterized by cognitive decline, functional impairment, and a range of behavioral and psychological symptoms (1). Among these, agitation is one of the most prevalent and distressing manifestations, affecting up to 90% of individuals with dementia over the course of their illness (2). Agitation encompasses a spectrum of behaviors, including physical aggression, physical non-aggressive behaviors (e.g., pacing, restlessness), and verbal agitation (e.g., screaming, repetitive questioning) (3). These behaviors are associated with accelerated cognitive decline, reduced quality of life for people with dementia, increased caregiver burden, and earlier institutionalization (4, 5).

Despite the high prevalence and clinical impact of agitation, the efficacy and safety of pharmacological treatments remain limited. Antipsychotic medications, commonly prescribed for agitation, are associated with significant adverse effects, including sedation, extrapyramidal symptoms, cerebrovascular events, and increased mortality (6, 7). Consequently, current clinical guidelines recommend non-pharmacological interventions as the first-line approach for managing agitation in dementia (8). These interventions aim to address underlying unmet needs—such as boredom, loneliness, or physical discomfort—that are hypothesized to trigger agitated behaviors (9).

Among non-pharmacological approaches, person-centered interventions tailored to the individual’s preferences, life history, and cognitive abilities have gained increasing attention (10). Such interventions encompass a diverse array of strategies, including Montessori-based activities, individualized music therapy, reminiscence therapy, multisensory stimulation, and structured activity programs tailored to the person’s remaining capabilities and interests (11–13). The underlying rationale is that personalization enhances engagement, positive affect, and a sense of meaning, thereby reducing agitation more effectively than generic social contact or usual care (9). A growing body of randomized controlled trials has demonstrated the efficacy of various personalized interventions in reducing agitation among individuals with dementia (14–16). However, the magnitude of the effect varies considerably across studies, and it remains unclear whether the efficacy differs by the subtype of agitated behaviors (e.g., physical aggression vs. verbal agitation), as most previous trials have reported aggregate agitation scores rather than subtype-specific outcomes.

Previous meta-analyses have examined the overall efficacy of non-pharmacological interventions for agitation in dementia, with findings supporting modest but significant benefits (14). However, these reviews have primarily focused on the average effect across all types of agitation and have largely overlooked the possibility that different behavioral subtypes may respond differently to personalized interventions. For example, physical aggression may be more closely linked to unmet physical needs (e.g., pain), whereas verbal agitation may be more responsive to social engagement (17). Understanding such differential effects is critical for clinical decision-making, as it could enable more targeted and cost-effective treatment selection. To date, no meta-analysis has systematically compared the effects of person-centered non-pharmacological interventions across different agitation subtypes.

Therefore, this meta-analysis aims to: (1) provide an updated pooled estimate of the overall efficacy of person-centered non-pharmacological interventions on agitation in individuals with dementia, serving as a foundational benchmark for comparability with prior reviews; and (2) conduct the first meta-analytic comparison of treatment effects across behavioral subtypes (physical aggressive, physical non-aggressive, and verbal agitation), which represents the primary novel contribution of this study. The findings of this study may inform clinical practice by providing evidence-based guidance on tailoring non-pharmacological interventions to the specific behavioral profiles of people with dementia.

## Methods

### Protocol and registration

This systematic review and meta-analysis was conducted in accordance with the Preferred Reporting Items for Systematic Reviews and Meta-Analyses (PRISMA) 2020 statement (18). The protocol was registered in the International Prospective Register of Systematic Reviews (PROSPERO) under registration number CRD420261363075.

### Eligibility criteria

Studies were included if they met the following criteria based on the PICOS framework: (1) Population: older adults (aged ≥ 60 years) with a clinical diagnosis of dementia of any type and severity; (2) Intervention: person-centered non-pharmacological interventions, defined as interventions that were tailored, personalized, or individualized based on at least one of the following participant characteristics: preferences, life history, cognitive abilities, social background, behavioral needs, or other relevant biological, psychological, or environmental factors (e.g., Montessori-based activities, individualized music therapy, robotic pet therapy, structured activity programs). Studies were considered eligible if they explicitly reported tailoring of the intervention to any of the above dimensions; no minimum number of tailored components was required; (3) Comparator: usual care, social contact, placebo, or active control interventions not specifically personalized; (4) Outcomes: agitation assessed by validated instruments, including but not limited to the Cohen-Mansfield Agitation Inventory (CMAI), the Chinese version of the CMAI (C-CMAI), the Cohen-Mansfield Agitation Inventory-Short Form (CMAI-SF), the Agitation Behavior Mapping Instrument (ABMI), or the Behavioral Pathology in Alzheimer’s Disease Rating Scale (BEHAVE-AD); and (5) Study design: randomized controlled trials (RCTs) published in English. Studies were excluded if they were non-randomized, quasi-experimental, case series, or if they did not report sufficient data (means, standard deviations, or sample sizes) to calculate effect sizes (19).

### Search strategy

A comprehensive literature search was conducted in four electronic databases: PubMed, Embase, Web of Science, and the Cochrane Central Register of Controlled Trials (CENTRAL), from database inception to March 2026. The search strategy combined terms related to dementia (e.g., “Alzheimer*,” “dementia”), agitation (e.g., “agitation,” “behavioral and psychological symptoms,” “BPSD”), non-pharmacological interventions (e.g., “person-centered,” “individualized,” “music therapy,” “Montessori,” “robotic pet”), and study design (e.g., “randomized controlled trial,” “RCT”). The full search strategy is provided in Supplementary materials. Additionally, reference lists of included studies and relevant systematic reviews were hand-searched to identify potentially eligible studies. Study authors were not contacted for missing or additional information; all analyses were conducted using data reported in the published articles.

### Study selection and data extraction

Two reviewers independently screened titles, abstracts, and full texts against the eligibility criteria. Disagreements were resolved through discussion or consultation with a third reviewer. Data extraction was performed independently by two reviewers using a standardized data extraction form, which captured: first author, year of publication, country, sample size, participant characteristics (age, sex, dementia type and severity), intervention details (type, frequency, duration), comparator description, agitation outcome measures (including instrument, timing, and reported data), and behavioral subtype data when available (physical aggressive, physical non-aggressive, and verbal agitation). For studies reporting agitation as subscale scores, data for each subtype were extracted separately.

### Risk of bias assessment

The methodological quality of the included randomized controlled trials was assessed independently by two reviewers using the Cochrane Risk of Bias 2.0 (RoB 2.0) tool (20). The RoB 2.0 tool evaluates five domains: (1) bias arising from the randomization process; (2) bias due to deviations from intended interventions; (3) bias due to missing outcome data; (4) bias in measurement of the outcome; and (5) bias in selection of the reported result. Each domain was rated as “low risk,” “some concerns,” or “high risk.” An overall risk-of-bias judgment was then assigned to each study based on the domain-level assessments. Disagreements were resolved by consensus or consultation with a third reviewer. No studies were excluded from the primary analysis based on their risk of bias rating; instead, the potential impact of bias was examined in sensitivity analyses.

### Outcome measures instruments

The primary outcome of interest was agitation, defined as inappropriate verbal, vocal, or motor activity not attributable to apparent needs or confusion (21). Across the included studies, agitation was assessed using various validated instruments, including the Cohen-Mansfield Agitation Inventory (CMAI) (21), the Chinese version of the CMAI (C-CMAI) (22), the Agitation Behavior Mapping Instrument (ABMI) (21), and the Behavioral Pathology in Alzheimer’s Disease Rating Scale (BEHAVE-AD) (23). Of note, the C-CMAI (22) is a validated Chinese translation of the CMAI; the study that used it was published in an English-language journal and thus met our inclusion criteria. For the BEHAVE-AD, the “aggressiveness” subscale was used to capture agitation-related outcomes. The specific instruments used in each included study are summarized in Table 1. When available, outcomes were further categorized into behavioral subtypes: (1) physical aggressive agitation; (2) physical non-aggressive agitation; and (3) verbal agitation, based on the classification framework proposed by Cohen-Mansfield (23).

**Table 1:** Characteristics of included studies.

Study (year)	Country	Sample size (I/C)	Dementia severity	Intervention	Control	Outcome measure	Intervention duration
van der Ploeg (2013) (24)	Australia	44/44	Severe (MMSE 6)	Montessori-based personalized activities	Non-personalized social interaction	ABMI (observation)	30 min, twice/week, 4 weeks
Moyle (2017) (25)	Australia	138/137	Moderate to severe	PARO robotic pet	Usual care	CMAI-SF	15 min, 3 times/week, 10 weeks
Ballard (2020) (26)	UK	404/443	Moderate to severe (CDR ≥ 1)	WHELD program	Usual care	CMAI	9 months
Sakamoto (2013) (27)	Japan	13/13	Severe (CDR = 3)	Individualized music therapy	No music	BEHAVE-AD (aggressiveness)	30 min, once/week, 10 weeks
Lin (2011) (28)	Taiwan	49/51	Moderate (MMSE 13.8)	Group music therapy	Usual activity	C-CMAI	30 min, twice/week, 6 weeks
Cohen-Mansfield (2012) (29)	USA	89/36	Advanced (MMSE 8.1)	TREA individualized intervention	Placebo (education)	ABMI	2 weeks (during peak agitation hours)

I/C, Intervention/Control; MMSE, Mini-Mental State Examination; CDR, Clinical Dementia Rating; CMAI, Cohen-Mansfield Agitation Inventory; CMAI-SF, Cohen-Mansfield Agitation Inventory-Short Form; C-CMAI, Chinese version of the Cohen-Mansfield Agitation Inventory; ABMI, Agitation Behavior Mapping Instrument; BEHAVE-AD, Behavioral Pathology in Alzheimer’s Disease Rating Scale; WHELD, Wellbeing and Health for People with Dementia; TREA, Treatment Routes for Exploring Agitation.

### Statistical analysis

All meta-analyses were performed using Review Manager version 5.4 (RevMan, Cochrane Collaboration, London, UK). Given the use of different agitation measurement instruments across studies, the standardized mean difference (SMD) with 95% confidence intervals (CIs) was chosen as the effect size metric. Hedge’s g was used as the SMD estimator to correct for small sample bias. A random-effects model was applied *a priori* to account for anticipated clinical and methodological heterogeneity across studies. Statistical heterogeneity was assessed using the *I*^2^ statistic, with values of 25, 50, and 75% considered as low, moderate, and high heterogeneity, respectively. For the primary meta-analysis of overall agitation, all included studies reporting total agitation scores were pooled. Statistical significance was set at a two-tailed *p*-value <0.05.

### Sensitivity analyses and publication bias

Subgroup analyses were conducted to examine whether treatment effects differed by behavioral subtypes of agitation. Based on the conceptual framework of agitation syndromes (21), studies were categorized into three subtypes when data were available: (1) physical aggressive agitation; (2) physical non-aggressive agitation; and (3) verbal agitation. For each subtype, separate meta-analyses were performed, and the between-subgroup differences were assessed using interaction tests.

Sensitivity analyses were performed to assess the robustness of the findings by: (1) excluding studies with a high risk of bias (if any); (2) excluding studies with high attrition rates; and (3) using a fixed-effects model instead of a random-effects model. Publication bias was planned to be assessed using funnel plots if at least 10 studies were included.

## Results

### Study selection

**Figure 1:**
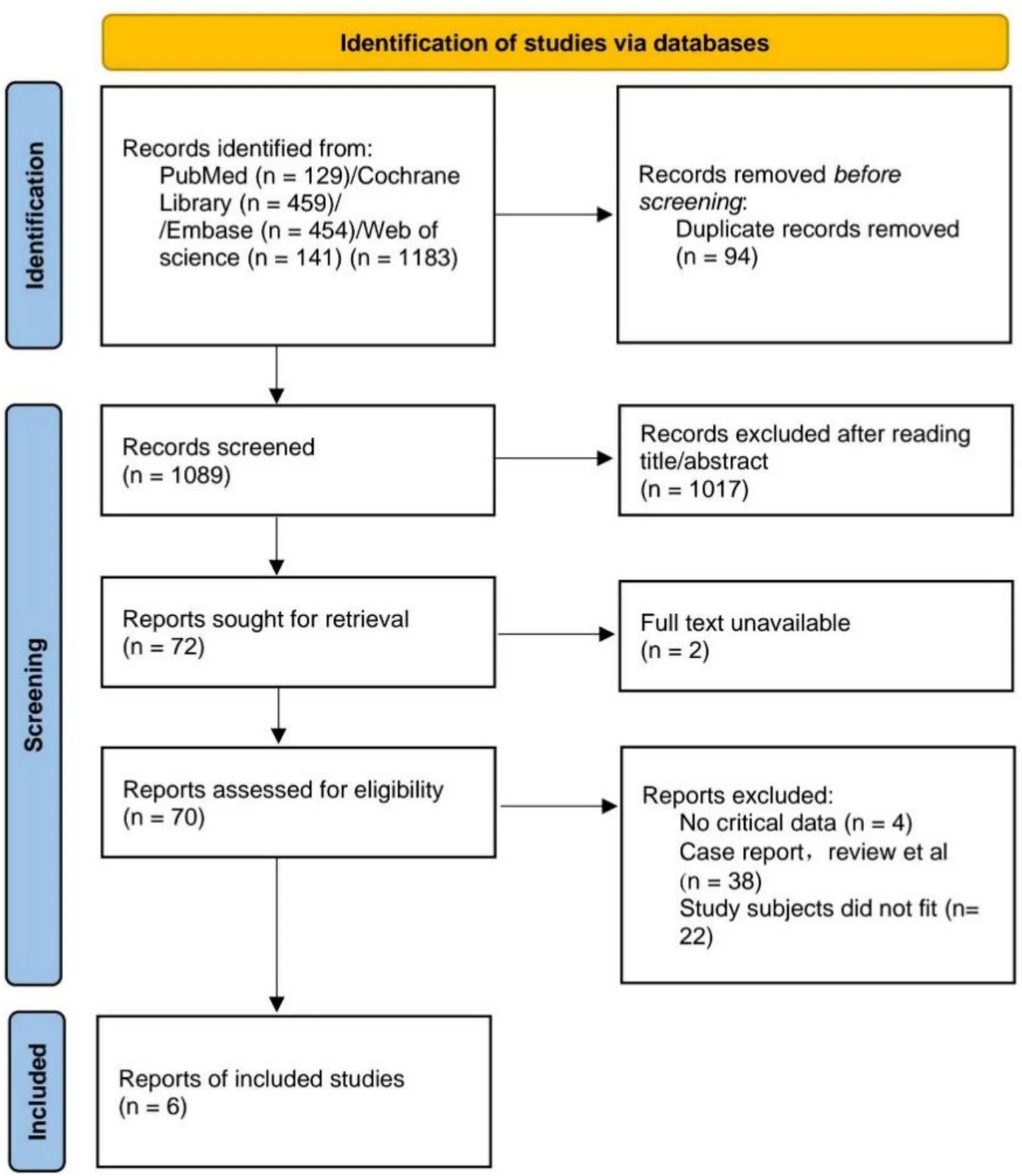
PRISMA flow diagram of study selection.

The literature search yielded a total of 1,183 records across the four electronic databases (PubMed: 129; Cochrane Library: 459; Embase: 454; Web of Science: 141). After removing 94 duplicate records, 1,089 articles were screened by title and abstract. Of these, 1,017 articles were excluded as they did not meet the eligibility criteria. The full texts of the remaining 72 articles were assessed for eligibility, of which two were unavailable. Of the 70 full-text articles reviewed, 64 were excluded during this full-text screening stage based on the following predefined criteria applied sequentially: non-RCT study design (case reports, reviews, or quasi-experimental studies) (*n* = 38), study population not meeting inclusion criteria (*n* = 22), and lack of critical outcome data (*n* = 4). No other exclusion criteria were applied at this stage. Ultimately, six randomized controlled trials met the inclusion criteria and were included in the quantitative synthesis (24–29). The study selection process is illustrated in Figure 1.

### Study characteristics

The characteristics of the six included randomized controlled trials (24–29) are summarized in Table 1. All studies were published between 2010 and 2020 and were conducted in Australia (*n* = 2), the United Kingdom (*n* = 1), the United States (*n* = 1), Japan (*n* = 1), and Taiwan (*n* = 1). The total number of participants across the six studies was 846, with individual study sample sizes ranging from 26 to 553. All participants had a clinical diagnosis of dementia, with the majority residing in long-term care facilities. The mean age of participants ranged from 81 to 86 years across studies, and the proportion of female participants ranged from 53 to 81%. Dementia severity varied, with most studies including participants with moderate to severe dementia as assessed by the Mini-Mental State Examination or Clinical Dementia Rating.

To systematically evaluate how each intervention operationalized person-centered principles, we mapped the intervention components of each included study to the core person-centered care framework proposed (10), which includes knowing the person, identifying individual needs and preferences, promoting meaningful activities and social interaction, and providing a supportive physical environment. This mapping is presented in Supplementary Table S1.

### Risk of bias assessment

**Figure 2:**
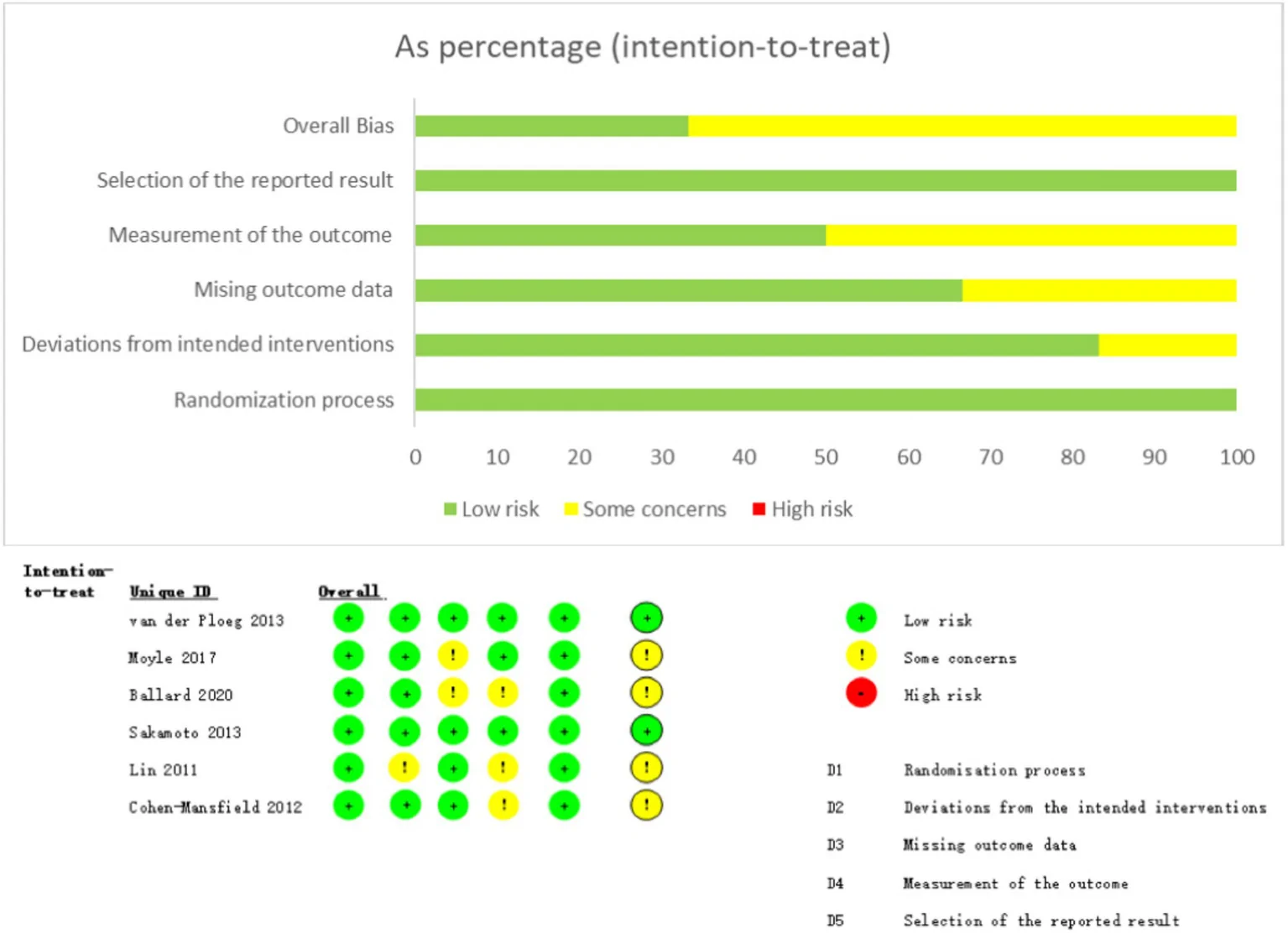
Risk of bias assessment summary (RoB 2.0).

The methodological quality of the six included randomized controlled trials was assessed using the Cochrane Risk of Bias 2.0 (RoB 2.0) tool (20). The results of the risk of bias assessment are summarized in Figure 2. Two studies were rated as having low risk of bias overall (24, 27), while four studies were rated as having some concerns (25, 26, 28, 29). No study was rated as high risk. The most common concerns across studies were related to deviations from intended interventions and measurement of the outcome, which is inherent to non-pharmacological intervention trials where blinding of participants and personnel is often not feasible. All studies demonstrated adequate randomization processes and low risk of selective reporting.

### Overall agitation

**Figure 3:**
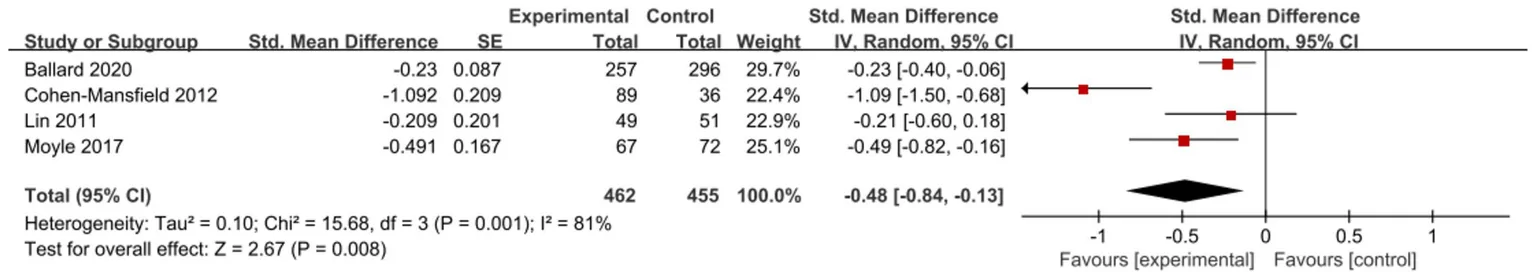
Forest plot for overall agitation. Data are presented as SMDs with 95% CIs; the diamond represents the pooled effect estimate. Negative values indicate a reduction in agitation favoring the intervention.

Four studies (*n* = 4) (25, 26, 28, 29) reported total agitation scores and were included in the meta-analysis for overall agitation. The pooled results showed that person-centered non-pharmacological interventions significantly reduced overall agitation compared with control conditions, with a standardized mean difference (SMD) of −0.48 (95% confidence interval [CI]: −0.84 to −0.13, *p* = 0.008). However, substantial heterogeneity was observed across studies (*I*^2^ = 81%, *p* = 0.001), indicating considerable variability in the effect sizes. A random-effects model was therefore applied. Directionally, negative standardized mean differences (SMDs) uniformly favor the intervention group across all analyses, whereas positive values favor the comparator. The forest plot for overall agitation is presented in Figure 3.

### Subgroup analysis by behavioral subtype

#### Physical aggressive agitation

**Figure 4:**
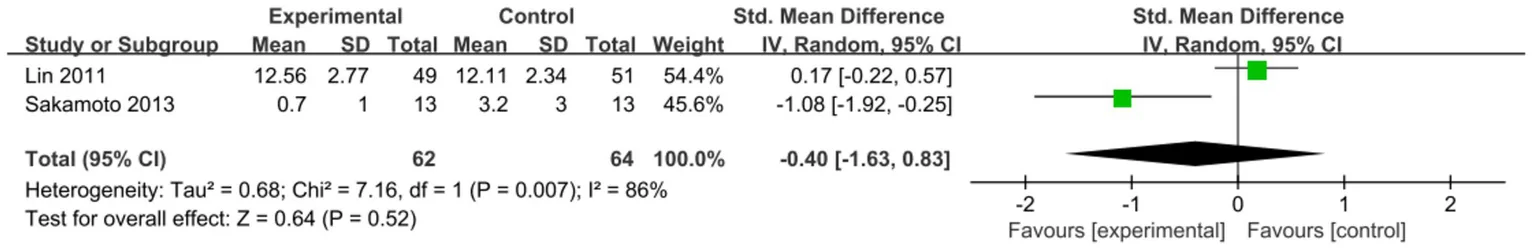
Forest plot for physical aggressive agitation.

Two studies (*n* = 2) reported outcomes for physical aggressive agitation (27, 28). The pooled SMD was −0.40 (95% CI: −1.63 to 0.83, *p* = 0.52), indicating no significant effect of the interventions on physically aggressive behaviors. Substantial heterogeneity was detected (*I*^2^ = 86%, *p* = 0.007). The forest plot for physical aggressive agitation is presented in Figure 4.

#### Physical non-aggressive agitation

**Figure 5:**
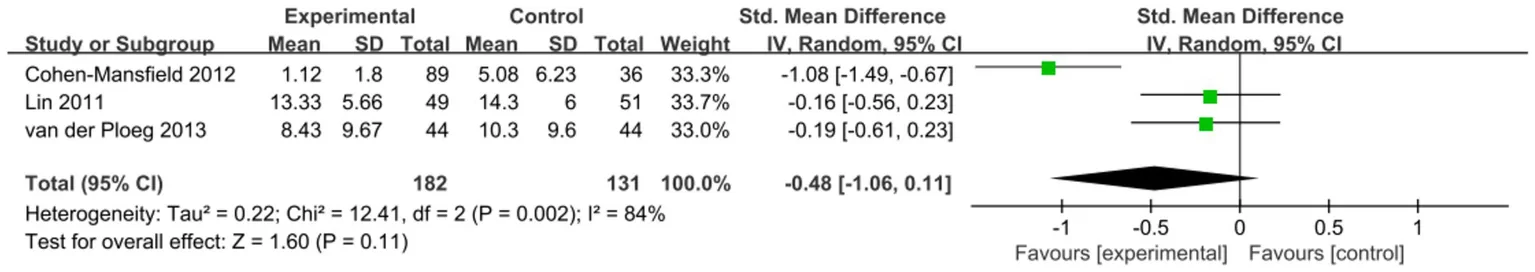
Forest plot for physical non-aggressive agitation. Data are presented as SMDs with 95% CIs; the diamond represents the pooled effect estimate. Negative values indicate a reduction in agitation favoring the intervention.

Three studies (*n* = 3) reported outcomes for physical non-aggressive agitation (24, 28, 29). The pooled SMD was −0.48 (95% CI: −1.06 to 0.11, *p* = 0.11), indicating a non-significant reduction in physical non-aggressive behaviors. High heterogeneity was observed (*I*^2^ = 84%, *p* = 0.002). The forest plot for physical non-aggressive agitation is presented in Figure 5.

#### Verbal agitation

**Figure 6:**
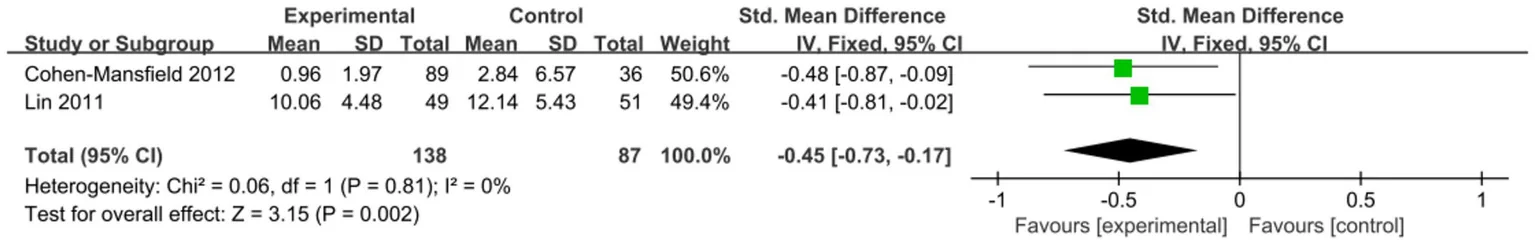
Forest plot for verbal agitation. Data are presented as SMDs with 95% CIs; the diamond represents the pooled effect estimate. Negative values indicate a reduction in agitation favoring the intervention.

Two studies (*n* = 2) reported outcomes for verbal agitation (28, 29). The pooled SMD was −0.45 (95% CI: −0.73 to −0.17, *p* = 0.002), indicating a statistically significant reduction in verbal agitation following person-centered non-pharmacological interventions. No heterogeneity was observed (*I*^2^ = 0%, *p* = 0.81), and a fixed-effects model was therefore applied. The forest plot for verbal agitation is presented in Figure 6.

### Publication bias

Publication bias was not formally assessed due to the limited number of included studies (*n* = 6 for the primary analysis; fewer than 10 studies for each subgroup), as funnel plot asymmetry tests have low statistical power under these conditions. Funnel plots were therefore not generated.

## Discussion

This meta-analysis synthesized evidence from six randomized controlled trials to evaluate the efficacy of person-centered non-pharmacological interventions for agitation in individuals with dementia, with a particular focus on differential effects across behavioral subtypes. The main findings demonstrated that such interventions significantly reduced overall agitation compared with control conditions (SMD = −0.48, 95% CI: −0.84 to −0.13, *p* = 0.008). Subgroup analyses revealed that the beneficial effect was primarily driven by improvements in verbal agitation (SMD = −0.45, 95% CI: −0.73 to −0.17, *p* = 0.002), whereas reductions in physical non-aggressive agitation (SMD = −0.48, 95% CI: −1.06 to 0.11, *p* = 0.11) and physical aggressive agitation (SMD = −0.40, 95% CI: −1.63 to 0.83, *p* = 0.52) did not reach statistical significance. Crucially, these subgroup analyses were based on only 2–3 studies each, which substantially limits statistical power; therefore, these subtype-specific findings should be viewed as exploratory and hypothesis-generating rather than conclusive. Substantial heterogeneity was observed across studies for overall agitation and physical agitation subtypes, but not for verbal agitation (*I*^2^ = 0%).

The overall effect size observed in this meta-analysis (SMD = −0.48) is consistent with previous systematic reviews that have examined non-pharmacological interventions for agitation in dementia. A comprehensive Health Technology Assessment review by Livingston et al. (14) reported that sensory and psychological interventions yielded modest but significant benefits for agitation, with effect sizes ranging from small to moderate. Similarly, a network meta-analysis by Watt et al. (15) found that multidisciplinary care, massage and touch therapy, and music combined with massage were among the most effective interventions for agitation, with standardized mean differences ranging from −0.49 to −0.71. Our pooled estimate of −0.48 is broadly consistent with this range, supporting the conclusion that person-centered non-pharmacological approaches confer clinically meaningful benefits.

However, the present study extends previous work by demonstrating that the overall effect masks important differences across behavioral subtypes. While verbal agitation showed a robust and homogeneous response (SMD = −0.45, *I*^2^ = 0%), physical agitation subtypes exhibited non-significant pooled effects with substantial heterogeneity. This pattern is consistent with the theoretical framework proposed by Cohen-Mansfield (9), which posits that different agitated behaviors may arise from distinct underlying unmet needs. Verbal agitation, such as screaming or repetitive questioning, may be more responsive to social engagement and personalized interaction, whereas physical agitation may be more closely linked to physical discomfort, pain, or sensory overload—needs that may not be adequately addressed by the interventions included in this analysis (17).

Substantial heterogeneity was observed for overall agitation (*I*^2^ = 81%) and for physical non-aggressive (*I*^2^ = 84%) and physical aggressive (*I*^2^ = 86%) agitation. Several factors may account for this heterogeneity. First, the interventions varied considerably across studies, ranging from Montessori-based activities and robotic pets to music therapy and structured individualized programs. Although all interventions were conceptually grounded in person-centered principles, their specific content, intensity, and duration differed (11). Second, the control conditions were not uniform, including usual care, non-personalized social interaction, and placebo education sessions, which may have influenced the magnitude of the observed treatment effects (29). Third, the included studies varied in terms of dementia severity, setting, and outcome measurement instruments, all of which can contribute to heterogeneity (30).

The findings of this meta-analysis have several important clinical implications. The significant reduction in overall agitation supports the use of person-centered non-pharmacological interventions as a first-line approach for managing agitation in dementia, consistent with current clinical guidelines (8). Moreover, the differential efficacy across behavioral subtypes suggests that clinicians should consider the specific presentation of agitated behaviors when selecting interventions. For people with dementia with prominent verbal agitation, personalized social engagement and sensory interventions may be particularly beneficial. In contrast, for people with dementia with physical agitation—especially aggressive behaviors—additional assessment and intervention targeting underlying physical discomfort, pain, or environmental factors may be necessary (17, 31).

The substantial heterogeneity observed for physical agitation subtypes highlights the need for more individualized assessment and intervention tailoring. A one-size-fits-all approach is unlikely to be effective for these behaviors. Structured approaches such as the Treatment Routes for Exploring Agitation (TREA) model (32), which systematically identifies unmet needs and matches interventions accordingly, may be particularly valuable for addressing physical agitation. In addition, the low heterogeneity and significant effect on verbal agitation suggest that even relatively simple personalized interventions—such as individualized music or one-on-one social interaction—can reliably reduce this distressing symptom, offering a feasible option for implementation in resource-limited settings (33).

This meta-analysis has several strengths. A key strength is that, to our knowledge, this is the first meta-analysis to systematically compare the effects of person-centered non-pharmacological interventions across different behavioral subtypes of agitation in dementia. The study employed rigorous methodology, including a comprehensive literature search, independent screening and data extraction, and risk of bias assessment using the updated RoB 2.0 tool. Additionally, all included studies were randomized controlled trials, which minimized the risk of selection bias and enhanced the internal validity of the pooled findings.

Several limitations should be acknowledged. First, the number of studies included in each subgroup analysis was small (two to three studies per subtype), which limits the precision of the pooled estimates and precludes formal publication bias assessment. Second, substantial heterogeneity was present for most outcomes, which could not be fully explored due to the limited number of studies. Third, the interventions varied widely in terms of content, intensity, and duration, which may limit the generalizability of the findings to specific intervention types. Fourth, the measurement instruments for agitation differed across studies, and although we used SMD to standardize effect sizes, differences in measurement properties may still introduce bias. Fifth, blinding of participants and personnel was not feasible in most non-pharmacological trials, which may have introduced performance and detection bias; however, all studies were rated as having low risk or some concerns, with no high-risk studies included. Finally, the majority of participants were recruited from long-term care facilities, limiting the applicability of findings to community-dwelling individuals with dementia.

The findings of this meta-analysis highlight several priorities for future research. A key priority is the need for large-scale, well-designed randomized controlled trials that specifically examine the effects of person-centered interventions on behavioral subtypes of agitation. Such trials should employ standardized outcome measures with validated subscales to facilitate meta-analytic pooling and improve comparability across studies (34). Beyond this, future studies should investigate the mechanisms underlying the differential effects across subtypes, including the role of pain, sensory processing, and social engagement in mediating treatment response (31, 35).

Another important direction concerns the optimal intensity, frequency, and duration of interventions, which remain unclear; dose–response studies are needed to establish evidence-based implementation protocols (36). Research should also explore the cost-effectiveness of personalized interventions relative to usual care, particularly given the resource constraints in long-term care settings (14). Given that the majority of existing studies have been conducted in institutional settings, future research should evaluate the effectiveness of person-centered interventions for community-dwelling individuals with dementia and their family caregivers. Equally important is the development and evaluation of scalable implementation strategies—such as training programs for family members or volunteers—which would facilitate broader dissemination of these effective interventions (37).

## Conclusion

This meta-analysis suggests that person-centered non-pharmacological interventions significantly reduce overall agitation in individuals with dementia, with the most consistent effects observed for verbal agitation across the included studies. However, these differential subtype effects should be interpreted with caution, as the subgroup analyses were based on only 2–3 studies each. The beneficial effects on physical agitation subtypes did not reach statistical significance, and substantial heterogeneity across studies underscores the need for more targeted approaches. These findings support the use of personalized interventions as a first-line strategy for managing agitation, particularly for verbal symptoms, while highlighting the importance of further research to optimize interventions for physical agitation. Clinicians should consider the specific behavioral presentation when selecting non-pharmacological approaches, and future studies should focus on developing and testing interventions tailored to the distinct underlying mechanisms of different agitation subtypes, with priority given to large-scale trials powered to detect subtype-specific effects.

## Data Availability

The original contributions presented in the study are included in the article/Supplementary material, further inquiries can be directed to the corresponding author/s.
