# Supplementary material for: Person-centered non-pharmacological interventions for agitation in dementia: a meta-analysis with subgroup analysis by behavioral subtypes
Source: Front Neurol. 2026 Aug 10;17:1850987. doi: 10.3389/fneur.2026.1850987 (PMC13500237; doi:10.3389/fneur.2026.1850987)
Supplement: Supplementary file 1 [file Data_Sheet_1.DOCX]

**Supplementary Material 1: Detailed Search Strategies**

| **Database** | **Search Strategy** |
| --- | --- |
| **PubMed** | ("Dementia"[Mesh] OR "Alzheimer Disease"[Mesh] OR dementia[tiab] OR alzheimer*[tiab] OR "vascular dementia"[tiab])  AND  ("Nonpharmacological interventions"[Mesh] OR "non-pharmacological intervention"[tiab] OR "non pharmacological intervention"[tiab] OR "patient-centered care"[Mesh] OR "patient centered"[tiab] OR "patient-centred"[tiab] OR "individualized intervention"[tiab] OR "personalized intervention"[tiab] OR "tailored intervention"[tiab] OR "multicomponent intervention"[tiab] OR "psychosocial intervention"[tiab] OR "music therapy"[Mesh] OR "aromatherapy"[Mesh] OR "exercise"[Mesh] OR "cognitive stimulation"[tiab])  AND  ("Agitation"[Mesh] OR "Psychomotor Agitation"[Mesh] OR agitation[tiab] OR "behavioral symptoms"[tiab] OR "behavioural symptoms"[tiab] OR "neuropsychiatric symptoms"[tiab] OR "NPS"[tiab] OR "challenging behavior"[tiab] OR "disturbing behavior"[tiab])  AND  ("randomized controlled trial"[pt] OR "controlled clinical trial"[pt] OR randomized[tiab] OR randomised[tiab] OR placebo[tiab] OR "clinical trial"[tiab] OR RCT[tiab]) |
| **Embase** | ('dementia'/exp OR 'dementia':ti,ab OR 'alzheimer disease'/exp OR 'alzheimer*':ti,ab OR 'vascular dementia':ti,ab) AND ('non pharmacological intervention'/exp OR 'non pharmacological intervention':ti,ab OR 'nonpharmacological intervention':ti,ab OR 'patient-centered care'/exp OR 'patient centered':ti,ab OR 'patient-centred':ti,ab OR 'individualized intervention':ti,ab OR 'personalized intervention':ti,ab OR 'tailored intervention':ti,ab OR 'multicomponent intervention':ti,ab OR 'psychosocial intervention':ti,ab OR 'music therapy'/exp OR 'aromatherapy'/exp OR 'exercise'/exp OR 'cognitive stimulation':ti,ab) AND ('agitation'/exp OR 'psychomotor agitation'/exp OR 'agitation':ti,ab OR 'behavioral symptoms':ti,ab OR 'behavioural symptoms':ti,ab OR 'neuropsychiatric symptoms':ti,ab OR 'nps':ti,ab OR 'challenging behavior':ti,ab OR 'challenging behaviour':ti,ab) AND ('randomized controlled trial'/exp OR 'controlled clinical trial'/exp OR 'randomized':ti,ab OR 'randomised':ti,ab OR 'placebo':ti,ab OR 'clinical trial':ti,ab OR 'rct':ti,ab) |
| **Cochrane Library** | ("Dementia" OR "Alzheimer Disease" OR dementia OR alzheimer* OR "vascular dementia") AND ("Nonpharmacological interventions" OR "non-pharmacological intervention" OR "non pharmacological intervention" OR "patient-centered care" OR "patient centered" OR "patient-centred" OR "individualized intervention" OR "personalized intervention" OR "tailored intervention" OR "multicomponent intervention" OR "psychosocial intervention" OR "music therapy" OR "aromatherapy" OR "exercise" OR "cognitive stimulation") AND ("Agitation" OR "Psychomotor Agitation" OR agitation OR "behavioral symptoms" OR "behavioural symptoms" OR "neuropsychiatric symptoms" OR NPS OR "challenging behavior" OR "disturbing behavior") AND ("randomized controlled trial" OR "controlled clinical trial" OR randomized OR randomised OR placebo OR "clinical trial" OR RCT) |
| **Web of Science** | ("Dementia" OR dementia OR alzheimer*) AND ("individualized intervention" OR "personalized intervention" OR "tailored intervention" OR "patient-centered care" OR "patient centred" OR "person centered care") AND ("Agitation" OR agitation OR "neuropsychiatric symptoms" OR NPS) AND ("randomized controlled trial" OR RCT OR randomized OR randomised) |
