# Supplementary material for: Person-centered non-pharmacological interventions for agitation in dementia: a meta-analysis with subgroup analysis by behavioral subtypes
Source: Front Neurol. 2026 Aug 10;17:1850987. doi: 10.3389/fneur.2026.1850987 (PMC13500237; doi:10.3389/fneur.2026.1850987)
Supplement: Supplementary file 2 [file Table_1.DOCX]

Supplementary Table S1 Mapping of included interventions to person-centered care principles

| Study | Intervention | Person-Centered Principle Applied | Operationalization Details |
| --- | --- | --- | --- |
| van der Ploeg (2013) | Montessori-based personalized activities | Knowing the person; Promoting meaningful activities | Activities tailored to individual's life history, remaining abilities, and interests; one-to-one interaction based on Montessori principles of autonomy and meaningful roles |
| Moyle (2017) | PARO robotic pet | Identifying individual needs and preferences | Responsive to patient's verbal and touch interactions; individualized engagement based on the patient's affective responses during each session |
| Ballard (2020) | WHELD program | All principles (comprehensive) | Person-centered assessment informing individualized care plans; includes meaningful activities, social interaction, and environmental modification |
| Sakamoto (2013) | Individualized music therapy | Identifying needs; Promoting meaningful activities | Music selections based on the patient's past preferences, cultural background, and life history; delivered in a one-to-one setting |
| Lin (2011) | Group music therapy | Promoting meaningful social interaction | Group singing and instrumental play; focused on familiar songs from participants' youth; facilitated social engagement among peers |
| Cohen-Mansfield (2012) | TREA individualized intervention | Knowing the person; Identifying unmet needs | Systematic assessment of triggers and underlying needs (e.g., pain, boredom, loneliness) followed by tailored activities (e.g., music, social contact, simulated presence) |
